# Stromal regulatory T-cells are associated with a favourable prognosis in gastric cancer of the cardia

**DOI:** 10.1186/1471-230X-9-65

**Published:** 2009-09-04

**Authors:** Matthias Haas, Arno Dimmler, Werner Hohenberger, Gerhard G Grabenbauer, Gerald Niedobitek, Luitpold V Distel

**Affiliations:** 1 Department of Radiation Oncology, Friedrich-Alexander-University of Erlangen-Nuremberg, Erlangen, Germany; 2 Institute for Pathology, Friedrich-Alexander-University of Erlangen-Nuremberg, Erlangen, Germany; 3 Departement of Surgery, Friedrich-Alexander-University of Erlangen-Nuremberg, Erlangen, Germany; 4 Institute for Pathology, St. Vincentiuskliniken Karlsruhe, Karlsruhe, Germany; 5 Institute for Pathology, Sana Klinikum Lichtenberg and Unfallkrankenhaus Berlin, Berlin, Germany

## Abstract

**Background:**

Recent evidence suggests that CD4^+^CD25^+^FoxP3^+ ^regulatory T-cells (Treg) may be responsible for the failure of host anti-tumour immunity by suppressing cytotoxic T- cells. We assessed the prognostic significance of tumour infiltrating lymphocytes (TIL) in intestinal-type gastric cardiac cancer.

**Methods:**

Tumour infiltrating lymphocyte (TIL) subsets and tumour infiltrating macrophages (TIM) were investigated in 52 cases using tissue microarrays. The interrelationship between the cell populations (CD3+, CD8+, CD20+, CD68+, GranzymeB+, FoxP3+) in different compartments and NED-survival was investigated (median follow-up time: 61 months).

**Results:**

Intraepithelial infiltration with TIL and TIM including Treg was generally low and not related to NED-survival. However, patients with large numbers of FoxP3^+ ^Treg in the tumour stroma (>125.9 FoxP3^+^TILs/mm^2^) had a median survival time of 58 months while those with low FoxP3^+ ^TIL counts (<125.9 FoxP3^+^TILs/mm^2^) had a median survival time of 32 months (p = 0.006). Patients with high versus low stromal CD68^+^/FoxP3^+ ^cell ratios in primary tumour displayed median survivals of 32 and 55 months, respectively (p = 0.008).

**Conclusion:**

Our results suggest that inflammatory processes within the tumour stroma of gastric intestinal-type adenocarcinomas located at the gastric cardia may affect outcome in two ways. Tumour-infiltrating macrophages are likely to promote carcinogenesis while large numbers of Treg are associated with improved outcome probably by inhibiting local inflammatory processes promoting carcinogenesis. Thus, inhibition of Treg may not be a feasible treatment option in gastric adenocarcinoma.

## Background

Gastric carcinoma is the third most common gastrointestinal tumour type in developed countries [1]. Similarities in risk-factors for adenocarcinoma of the gastric cardia and the oesophagus [2] together with the stable incidence of cardiac gastric cancer despite an overall decline of gastric malignancies [3] suggest that these tumours should be separated from distally located gastric adenocarcinomas [4].

Escape from immunosurveillance is a fundamental step in cancer development. There is accumulating evidence indicating that CD4^+^CD25^+^FoxP3^+ ^regulatory T-cells (Treg) are able to induce tolerance to self-antigens and may also inhibit anti-tumour immune response. Tregs can inhibit effector T-cells, suppress the antigen presenting function and promote the immunosuppressive function of dendritic cells, monocytes and macrophages [5,6].

The prognostic significance of tumour infiltrating lymphocytes (TIL) in cancer therapy is not fully understood. A beneficial prognostic effect of increased lymphocyte infiltration has been shown for various tumour types. In ovarian carcinoma both intratumoural CD3^+ ^and CD8^+ ^TIL were associated with improved survival [7,8] while Treg were identified as a significant negative prognostic factor [9]. An inverse effect was observed in anal cancer where low amounts of CD3^+ ^and cytotoxic TIL were prognostically favourable, while Treg presence had no influence on prognosis [10]. A recent investigation of gastric malignancies found no correlation between the number of Treg and patient survival, but emphasized the significance of their distribution pattern [11].

We investigated the prognostic value of infiltration by various TIL-subgroups and macrophages including Treg in adenocarcinoma of the cardia in both primary tumour and draining lymph nodes.

## Methods

### Patients

Between 1993 and 2004, 135 patients with gastric adenocarcinoma of the cardia underwent evaluation and primary surgical treatment at the Erlangen University Hospital. A subgroup of 52 patients was enrolled in the present study according to the following criteria: All patients were required to have invasive gastric adenocarcinoma of the intestinal subtype according to the Laurén classification without distant metastases at presentation. Patients with oesophageal Barrett's adenocarcinoma were excluded. An exclusively surgical treatment regimen with complete resection (R0) was performed. No patient received (neo)adjuvant radiotherapy or chemotherapy. Staging was recorded according to the International Union against Cancer system (UICC 2002). We gathered data concerning recurrence and survival both from our university hospital, the patients' physicians and the respective residents registration office. The minimum follow-up time was 36 months; the mean follow-up time was 71.2 months (median 61.0 months) with a 95% confidence interval of 54.7 to 57.8 months. The use of the tissue sections and the patients' survival data following patient consent was approved by the Friedrich-Alexander University Erlangen-Nuremberg ethics committee.

### Tissue microarray and immunohistochemistry

**Figure 1:**
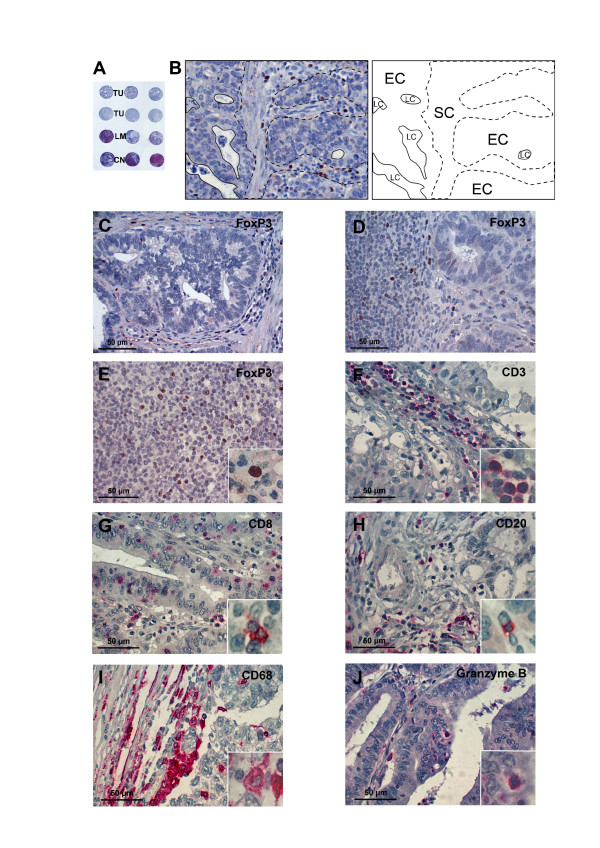
**Tissue microarrays**. (A) Tissue microarray, tumor punches [TU], metastatic lymph node punches [LM] and control lymph node punches stained for CD3. (B) Primary tumor side. By means of a semi-automatic image processing software (Biomas, Erlangen, Germany), the count of positively stained cells was set into relation to the surface of the epithelial compartment [EC] (after exclusion of the luminal compartment [LC]) and the stromal compartment [SC], respectively. (C) FoxP3^+ ^TILs in primary tumor site and the stromal compartment (D) FoxP3^+ ^cells in the lymph node (E) FoxP3^+ ^cells in control lymph node (F) CD3^+ ^TILs in primary tumor site (G) CD8^+ ^TILs in primary tumor site (H) CD20^+ ^TILs in primary tumor site (I) CD68^+ ^macrophages in primary tumor site (J) Granzyme B^+ ^TILs in primary tumor site.

474 core biopsies from primary tumour, uninvolved draining lymph nodes and, if applicable, metastatic nodes were processed into tissue microarrays (TMA) (Figure 1). As guide for microarray sampling, representative areas on standard H.E.-stained sections were marked by an experienced GI-pathologist (A.D.). A manual tissue arrayer (MTA-1, Beecher Instruments, Inc., Sun Prairie, WI, USA) was used for TMA construction. From each marked region three 1.0 mm cores were collected resulting in a total of six samples from the primary tumour site, three samples from uninvolved lymph nodes and three samples from metastatic lymph nodes, respectively. Among patients with positive lymph nodes, sufficient metastatic tissue for further processing was available in 22 of 28 cases. The samples punched off from the donor blocks were arrayed in a recipient paraffin block. TMA slides were stained as described before [10] using CD3, CD8, CD20, CD68, anti-Granzyme B antibodies (all from Dako, Hamburg, Germany), and a polyclonal anti-FoxP3 reagent (Abcam, Cambridge, United Kingdom).

### Quantification of TIL

Evaluation of the immunostained microarrays was performed using a standard light microscope at a magnification of ×200, a CCD-camera and a semi-automatic image processing software (Biomas, Erlangen, Germany). Labelled lymphocytes and macrophages were counted. In each image surface areas of the epithelial and the stromal compartments were calculated and glandular luminal areas were subtracted. Thus, it was possible to assess separately intraepithelial and stromal lymphocyte and macrophage infiltration both in the primary tumour site and lymphatic metastatic tissue (Figure 1). For each case and immune cell subset six images with most abundant infiltration were acquired both of intraepithelial and stromal compartments (mean total area per case determined for CD3: 0.466 mm^2^, mean epithelial surface area per case: 0.241 mm^2^, mean stromal surface area per case: 0.225 mm^2^). In stromal tissue, only areas adjacent to tumour infiltration were selected. In uninvolved lymph-nodes, two images were acquired per core adding to a total of six images per case.

### Statistical Analysis

Clinical variables evaluated were age, gender, UICC stage, T category and N category. Overall survival was defined as the period from surgery until death or end of follow-up. No-evidence-of-disease (NED) survival was evaluated as the period from surgery until evidence of recurrence (tumour recurrence, nodal recurrence or distant metastases). A univariate regression analysis of NED-survival was performed using Cox's proportional hazards model (SPSS, Chicago, IL, USA). For a multivariate analysis of NED-survival we adopted only variables being of significant impact in univariate analysis. Survival was analyzed by the Kaplan-Meier method [12]. The log-rank test [13] was used to compare survival curves between subgroups of patients. Median values of TIL/TIM-infiltration were used as cut-off.

## Results

### Study group

The patients' clinical and histological characteristics are depicted in Tables 1 and Tables 2. Overall survival and NED-survival rates of the study group were 53.7% and 63.7% at 5 years, respectively. Twelve patients died of unrelated diseases. Median overall survival and NED-survival time for all patients were 50 months (range, 46.4 - 70.8 months) and 41.5 months (range, 43.1 - 68.3 months), respectively.

**Table 1:** Patient characteristics: stage, grade and status.

		N (%)
Factor	All patients	52 (100)

Gender	Male	40 (76.9)

	Female	12 (23.1)

TNM stage (UICC 2002)	I	20 (39.2)

	II	19 (36.5)

	III	10 (19.2)

	IV	3 (5.8)

Grading	G 1	1 (2)

	G 2	29 (56)

	G 3	21 (40)

	G 4	1 (2)

T category	T 1	12 (23)

	T 2	32 (62)

	T 3	5 (10)

	T 4	3 (6)

N category	N 0	24 (46.2)

	N +	28 (53.8)

	N 1	22 (42.3)

	N 2	6 (11.5)

Any Relapse	Yes	17 (32.7)

	No	35 (67.3)

Local recurrence	Yes	5 (9.6)

	No	47 (90.4)

Distant Metastasis	Yes	14 (26.9)

	No	38 (73.1)

Status	Alive with NED	25 (48)

	Dead of disease	15 (29)

	Dead without disease	12 (23)

**Table 2:** Patient characteristics: follow-up and survival.

	Mean	Median	**95% C.I**.
Age (years)	64.6	67.0	61.5 - 67.7

Follow-up time (months)	71.2	61.0	54.7 - 57.8

Overall survival time (months)	58.6	50.0	46.4 - 70.8

NED-survival time (months)	55.7	41.5	43.1 - 68.3

Time to relapse (months)	23.8	17.0	13.8 - 33.7

### Distribution pattern of lymphocytes in gastric adenocarcinoma

Due to the clear border between the stromal and epithelial compartment in intestinal-type carcinomas, we evaluated the two compartments separately. A pronounced difference in TIL/TIM- infiltration between these stromal compartments was detected both at primary tumour site and in lymph node metastasis (Table 3).

**Table 3:** Descriptive statistics for TILs and macrophages in the primary tumour, metastatic lymph nodes and non affected lymph nodes for intraepithelial and stromal location.

Cells per mm^2^	Mean					
	**Primary tumour**		**Metastatic lymph node**			**Uninvolved lymph node**

	**epithelial**	**stromal**	**epithelial**	**stromal**	**lymphatic tissue**	

CD3	122.8	895.7	63.5	645.1	3710.1	7686.7

CD8	114.5	424.4	98.0	358.2	1071.9	2436.1

CD20	18.9	438.7	9.4	351.4	5612.7	n.d.

CD68	18.7	482.7	17.9	617.1	1457.8	506.4

FoxP3	14.3	187.5	12.1	90.2	532.0	337.0

Gran.B	3.0	39.8	0.8	42.3	89.4	46.9

						

**Cells per mm^2^**	**95% confidence interval of mean**					

	**Primary tumour**		**Metastatic lymph node**			**Uninvolved lymph node**

	**epithelial**	**stromal**	**epithelial**	**stromal**	**lymphatic tissue**	

CD3	71.2 - 174.5	714.6 - 1076.8	3.7 - 123.3	389.4 - 900.9	3149.5 - 4270.7	7088.1 - 8285.4

CD8	51.1 - 178.0	299.6 - 549.2	0 (-35.3) - 231.3	146.2 - 570.3	696.9 - 1447.0	2203.2 - 2669.0

CD20	0 (-0.1) - 37.9	249.7 - 627.7	0 (-2.1) - 20.9	180.9 - 521.8	4800.7 - 6424.8	n.d.

CD68	12.5 - 24.9	407.6 - 557.7	2.2 - 33.6	388.6 - 845.6	1051.8 - 1863.9	451.5 - 561.4

FoxP3	6.5 - 22.2	142.0 - 233.0	5.7 - 18.5	51.5 - 128.8	179.3 - 884.7	256.2 - 417.7

Gran.B	0.6 - 5.4	28.3 - 51.2	0 - 1.7	21.4 - 63.2	17.7 - 161.1	32.8 - 61.0

**Cells per mm^2^**	**Median**					

	**Primary tumor**		**Metastatic lymph node**			**Uninvolved lymph node**

	**epithelial**	**stromal**	**epithelial**	**stromal**	**lymphatic tissue**	

CD3	40.9	752.1	18.3	366.2	3625.3	7752.0

CD8	21.6	212.7	2.8	203.7	888.5	2433.4

CD20	0	146.7	0	242.8	5855.7	n.d.

CD68	11.5	480.9	2.3	487.5	1238.8	478.6

FoxP3	3.6	125.9	8.0	75.6	225.7	245.4

Gran.B	0	26.7	0	23.4	42.9	34.1

Numbers represent mean and median values for labelled cells per mm^2^. In addition, 95% confidence intervals of the mean are given.

**Figure 2:**
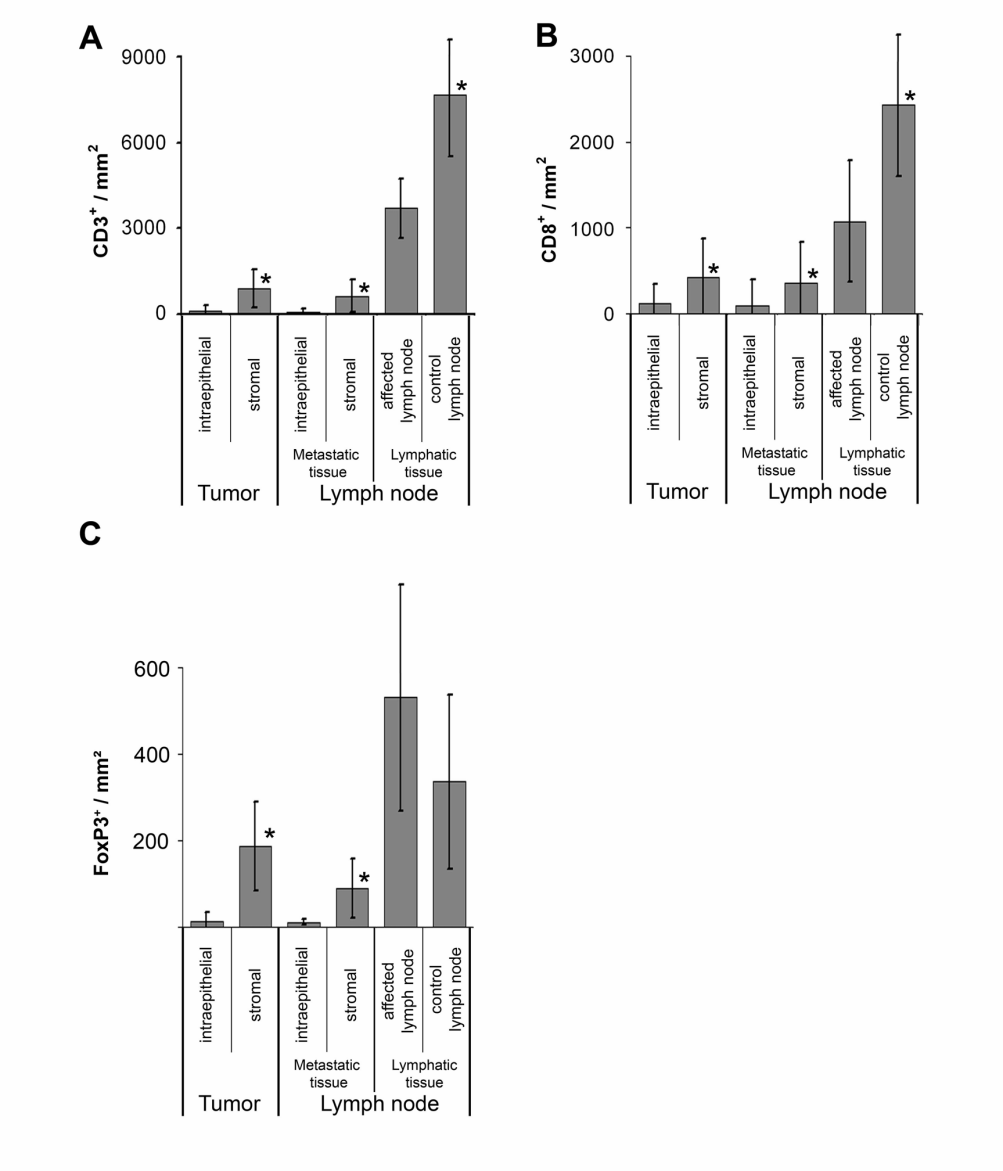
**Mean counts of TIL-subtypes in different compartments**. The counts are given per mm^2^. (A) CD3^+ ^counts (B) CD8^+ ^counts (C) FoxP3^+ ^counts. The asterisks (*) indicate that a statistical significant difference (p < 0.001 level) was observed between intraepithelial and stromal compartment of primary tumor and metastatic tissue of the lymph node, respectively, and between affected and uninvolved lymph node.

All TIL subsets and macrophages were found predominantly in stroma adjacent to epithelial tumour cell clusters while they occurred only infrequently between epithelial cells within the tumour cell nests (Figures 1 and 2).

The proportion of Tregs of total T cell counts was larger in areas of tumour infiltration (tumour stroma, affected lymph nodes) than in the control lymph nodes (Table 3).

### Prognostic significance of tumour infiltrating lymphocytes (TIL) and tumour infiltrating macrophages (TIM)

**Figure 3:**
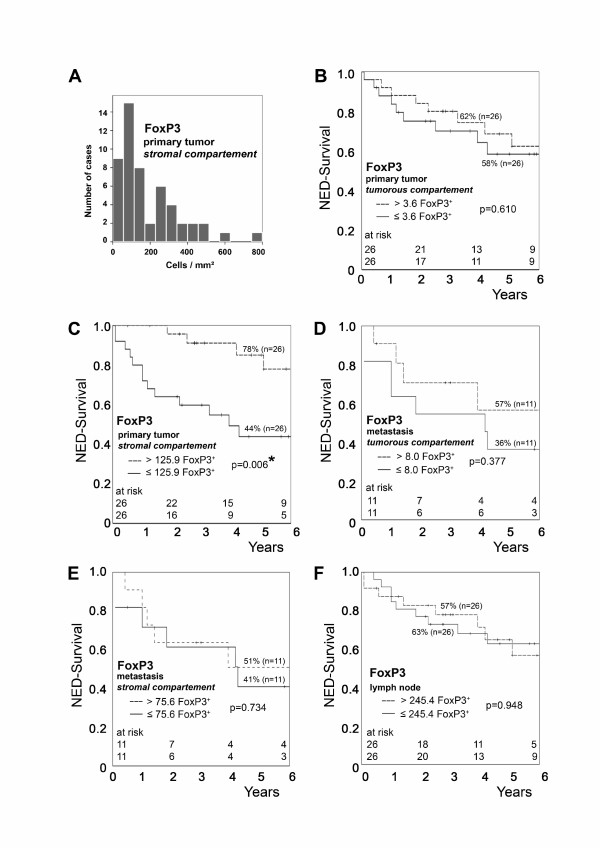
**Prognostic significance of FoxP3^+ ^lymphocytes in gastric adenocarcinoma of the cardia**. NED-survival rates in relation to the TIL infiltration were calculated by the Kaplan-Meier method and analyzed by the log-rank test. (A) Distribution of FoxP3^+ ^cell counts in stromal compartment of primary tumor. The abscissa shows the average number of intraepithelial FoxP3^+ ^TILs while the ordinate depicts the number of cases in each class. (B) Impact of FoxP3^+ ^TILs in epithelial compartment of primary tumor, (C) impact of FoxP3^+ ^TIL in stromal compartment of primary tumor, (D) impact of FoxP3^+ ^TILs in tumor compartment of metastasis, (E) impact of FoxP3^+ ^TILs in stromal compartment of metastasis, (F) impact of FoxP3^+ ^cells in lymph nodes.

FoxP3^+ ^TIL infiltration of the tumour stroma at the primary site was of pronounced prognostic significance: patients with high stromal FoxP3^+ ^TIL counts (>125.9 FoxP^+ ^TIL/mm^2^) had a median survival time of 58 months while those with low FoxP3^+ ^counts (<125.9 FoxP3^+ ^TIL/mm^2^) had a median survival time of 32 months (hazard ratio = 0.23; C.I. 0.08 - 0.72; p = 0.006; Figure 3C and Table 3). Considering the epithelial compartment of the primary tumour site only, none of the TIL subsets or macrophages had significant prognostic impact on NED-survival.

In multivariate analysis including stromal FoxP3^+ ^TIL, UICC-stage and nodal status, stromal Treg count was the only independent significant variable with impact on NED-survival (Table 4).

**Table 4:** Univariate and multivariate analysis of NED-survival according to Cox's proportional hazards model.

	Univariate Analysis			Multivariate Analysis		
**Variable**	**Hazard ratio**	**95% C.I**.	**P**	**Hazard ratio**	**95% C.I**.	**P**

Age, years (continuous)	1.01	0.96-1.06	0.78	-	-	-

Gender (female [n = 12] vs male [n = 40])	0.48	0.11-2.10	0.33	-	-	-

Stage (UICC II and higher [n = 32] vs. UICC I [n = 20])	3.72	1.07-12.98	0.04	2.00	0.42-9.09	0.39

Grade (3+4 [n = 22] vs. 1+2 [n = 30])	1.43	0.55-3.71	0.46	-	-	-

T category (T3/T4 [n = 8] vs. T1/T2 [n = 44])	2.48	0.80-7.68	0.11	-	-	-

N category (. N+ [n = 28] vs N0 [n = 24])	3.27	1.06-10.02	0.04	2.04	0.51-8.33	0.32

Number of metastatic lymph nodes (continuous)	1.35	1.17-1.56	<0.001	-	-	-

**TILs and macrophages in primary tumour site (high vs. low)**						

	**Hazard ratio**	**95% C.I**.	**P**	**Hazard ratio**	**95% C.I**.	**P**

**Intraepithelial**						

CD3+ TIL	0.76	0.29-1.98	0.58	-	-	-

CD8+ TIL	1.26	0.48-3.32	0.63	-	-	-

CD20+ TIL	2.05	0.79-5.32	0.14	-	-	-

CD68+ TIM	0.69	0.27-1.80	0.45	-	-	-

FoxP3+ TIL	0.78	0.30-2.03	0.61	-	-	-

Granzyme B+ TIL	0.72	0.21-2.53	0.61	-	-	-

**Stromal**				-	-	-

CD3+ TIL	0.86	0.33-2.23	0.76	-	-	-

CD8+ TIL	0.85	0.33-2.20	0.74	-	-	-

CD20+ TIL	0.80	0.31-2.08	0.65	-	-	-

CD68+ TIM	0.81	0.31-2.10	0.66	-	-	-

FoxP3+ TIL	0.23	0.08-0.72	**0.01**	0.25	0.08-0.78	**0.016**

Granzyme B+ TIL	0.87	0.33-2.29	0.78	-	-	-

Ratio stromal CD68 (primary tumour)/stromal FoxP3 (primary tumour)	4.02	1.31-12.36	**0.02**			

**TIL and macrophages in metastatic tissue (high vs. low)**						

**Intraepithelial**	**Hazard ratio**	**95% C.I**.	**P**			
			
CD3+ TIL	0.32	0.09-1.12	0.07			
			
CD8+ TIL	1.14	0.35-3.76	0.83			
			
CD20+ TIL	0.20	0.03-1.58	0.13			
			
CD68+ TIM	0.96	0.29-3.16	0.95			
			
FoxP3+ TIL	0.58	0.17-1.99	0.39			
			
Granzyme B+ TIL	0.88	0.19-4.09	0.87			
			
**Stromal**						
			
CD3+ TIL	0.61	0.19-2.03	0.42			
			
CD8+ TIL	1.16	0.35-3.81	0.81			
			
CD20+ TIL	0.51	0.15-1.75	0.28			
			
CD68+ TIM	1.30	0.40-4.28	0.66			
			
FoxP3+ TIL	0.81	0.25-2.69	0.74			
			
Granzyme B+ TIL	0.75	0.23-2.46	0.63			

**Lymphocytes and macrophages in uninvolved regional lymph node (high vs. low)**						

	**Hazard ratio**	**95% C.I**.	**P**			
			
CD3+	0.47	0.17-1.26	0.13			
			
CD8+	0.84	0.32-2.19	0.72			
			
CD20+						
			
CD68+	0.56	0.21-1.47	0.24			
			
FoxP3+	1.03	0.40-2.68	0.95			
			
Granzyme B+	0.35	0.12-0.99	**0.05**			

For multivariate Cox's proportional hazards model UICC-stage and nodal status were adopted as covariates. Median values were used as cut-off.

The ratio of stromal CD8^+ ^and FoxP3^+ ^cells was not associated with significant differences in survival. However, the stromal CD68^+^/FoxP3^+ ^cell ratio was of prognostic significance (Table 4). In survival analysis, patients with a high stromal CD68^+^/FoxP3^+ ^cell ratio (> 2.9) at the primary tumour site had a median survival of 32 months while those with a lower CD68^+^/FoxP3^+ ^cell ratio (< 2.9) had a median survival of 55 months (p = 0.008) (graph not shown). Considering the uninvolved regional lymph nodes alone, higher numbers of Granzyme B^+ ^cells were linked to a beneficial outcome (hazard ratio = 0.35; C.I. = 0.12 - 0.99; p = 0.05).

### Correlation between TIL/TIM and clinical factors

Early disease (UICC stage I) was associated with significantly better NED-survival rates compared to advanced disease (UICC II-IV) (hazard ratio = 3.72; C.I. = 1.07 - 12.08; p = 0.04). An increasing stromal FoxP3^+ ^TIL infiltration was inversely correlated with UICC-stage (Pearson's correlation coefficient, r = -0.40; p = 0.001), number of lymph node metastases (r = -0.36; p = 0.009) and N category in general (r = -0.36; p = 0.023). For other cell types, no such correlation could be found.

## Discussion

Tregs inhibit the function of effector cells [5,6]. Consequently, an inhibitory effect of Treg on anti-tumour immunity has been predicted. In agreement with this hypothesis, large numbers of tumour-infiltrating Treg were shown to be associated with poor outcome in ovarian carcinomas [9]. However, studies of other tumours have produced conflicting results. Thus, no prognostic effect of Treg was observed in a study of anal carcinomas [10]. In certain lymphomas, tumour-infiltrating Treg were associated with favourable prognosis [14]. A recent study of FoxP3^+ ^Treg in colorectal cancer also showed high Treg numbers to be associated with improved survival [15].

Studies on gastric carcinomas have also yielded conflicting results. Kono et al. found a higher percentage of Treg among PBMCs in patients with advanced gastric carcinoma. Higher numbers of Treg in PBMCs had an adverse effect on outcome, and numbers of Treg declined to normal values following curative resection [16]. Mizukami et al. found no effect of Treg counts on survival but showed that a diffuse dissemination of Treg was associated with poor survival while accumulation of Treg in the peritumoural region correlated with a more favourable outcome. But the former distribution pattern was associated with diffuse gastric cancers while the latter was predominantly found in intestinal type carcinomas [11]. Kawaida et al. found that tumour draining lymph nodes showed an increased prevalence of CD4^+^CD25^+ ^TIL as compared to control mesenteric nodes. However, no specific correlation between survival and intensity of Treg infiltration could be demonstrated [17]. These previous studies have analyzed TIL numbers in different compartments ranging from primary tumour to PBMC, thus making comparison of results difficult.

For these reasons, we have decided to focus on a morphologically homogeneous group of intestinal-type adenocarcinomas located at the gastric cardia. We have assessed the numbers of tumour-infiltrating immune cells in different tumour-related compartments in relation to outcome parameters. We show firstly that the presence of various TIL subsets including Treg, cytotoxic T-cells as well as tumour-infiltrating macrophages within the epithelial compartment had no significant influence on survival rates. This is in contrast to our previous analysis of anal carcinomas [10]. This may be attributable to the differing growth patterns of the two tumour entities with anal carcinomas growing in a more solid fashion with less stromal support as compared to gastric adenocarcinoma. We identified only two parameters which were of prognostic significance. High numbers of Treg in the tumour stroma at the primary site were associated with improved prognosis while this was not the case for other tumour-related compartments. Furthermore, high numbers of macrophages in combination with low numbers of Treg in the tumour stroma indicated poor outcome. Moreover, we show that the number of Tregs as a proportion of total T-cells is higher in both in tumour stroma and in affected lymph nodes as compared to uninvolved lymph nodes. This enrichment of Tregs suggests that these cell are actively recruited to the tumour site and points to a role for Tregs in inhibiting local immune reactions by direct cell-to-cell contact [18].

On the face of it, these findings are unexpected and contrast previous studies of ovarian carcinoma [7-9,19]. Nevertheless, our findings parallel recent studies of Treg in colorectal cancer [15]. It is important to keep in mind that Treg exert their function via a direct membrane contact mechanism [18]. This might explain the observation that a larger proportion of T cells are regulatory T cells both in the tumor stroma and the affected lymph nodes as compared to the control lymph nodes.

Chronic inflammation, either related to H. pylori infection or to autoimmune gastritis, is considered the major risk factor for the development of gastric cancer of intestinal type. This is probably also true for gastric adenocarcinoma located at the cardia [20]. Development of chronic H. pylori gastritis and progression to gastric atrophy and preneoplastic lesions has been shown to be associated with the development of a Th1-predominant local immune reaction in mouse experiments [21]. Thus, the presence of large numbers of local Treg may well help to dampen these local inflammatory responses. This notion is also supported by the observation that gastric Treg can suppress H. pylori-induced T cell proliferation and IFN-gamma production [22]. It is speculated that Treg may inhibit local effector T-cells contributing to gastric carcinogenesis. A possible mechanism may be that the effect of Treg is mediated via a down-regulation of harmful proinflammatory cytokines known to promote malignancy [23-25]. The notion that Treg may exert an inhibitory effect on gastric cancer development is also in keeping with our observation that Treg are particularly prevalent in earlier stages of gastric carcinogenesis since any effect of these cells is likely to be greatest at this time point. Nonetheless, further functional studies will be necessary to elucidate the exact role of Treg in gastric cancer. Moreover, there is the emerging concept that not all FoxP3 positive cells might be natural Tregs with stable FoxP3 expression. Natural Treg cells might be detected by analyzing the Treg-cell specific demethylated region (TSDR) in tissue specimen [26].

We also find that a higher number of Granzyme B positive T cells in uninvolved lymph nodes is associated with a better outcome. Granzyme B is a key cytolytic mediator secreted by activated cytotoxic T cells. A large systemic number of activated cytotoxic T cells as represented by large numbers in uninvolved lymph nodes may be an indicator of an efficient systemic host anti-tumor immunity [27].

Moreover, we show that large numbers of tumour-associated macrophages in conjunction with small numbers of Treg are associated with poor prognosis. This is in agreement with a previous study [28]. It is now well accepted that tumour-associated macrophages promote tumour development for example through stimulating angiogenesis or modulating the plasticity of extracellular matrix [29]. Moreover, it has been shown recently that activated macrophages may activate Wnt signalling in gastric cancer epithelial cells via TNF-alpha thus contributing to tumour development [30].

## Conclusion

Our results suggest that inflammatory processes within the tumour stroma of intestinal-type adenocarcinoma of the gastric cardia may affect outcome in two ways. Tumour-infiltrating macrophages are likely to promote carcinogenesis as has been reported previously for various tumour models. By contrast, Treg may down-modulate local inflammatory processes which promote carcinogenesis. Thus, inhibition of Treg may not be a feasible treatment option in gastric adenocarcinoma.

## Competing interests

The authors declare that they have no competing interests.

## Authors' contributions

MH: conception and design, analysis and interpretation of data, drafting of manuscript. AD: analysis and interpretation of data. WH: conception and design. GGG: conception and design, revision of manuscript. GN: conception and design, revision of manuscript. LVD: conception and design, interpretation of data, drafting of manuscript. All authors read and approved the final manuscript.

## Pre-publication history

The pre-publication history for this paper can be accessed here:

http://www.biomedcentral.com/1471-230X/9/65/prepub
